# What Should Be the Roles of Conscious States and Brain States in Theories of Mental Activity?**

**DOI:** 10.4103/0973-1229.77429

**Published:** 2011

**Authors:** Donelson E. Dulany

**Affiliations:** Department of Psychology, University of Illinois, Urbana-Champaign, Champaign, IL 61820; ** *This paper is an elaboration and peer reviewed version of a Keynote Address for the International Seminar on Mind, Brain, and Consciousness, Thane College Campus, Thane, India, January 13, 2010*

**Keywords:** *Brain imaging*, *Consciousness*, *Deliberative*, *Evocative*, *Mentalism*, *Neural Networks*, *Phenomenal reports*, *Symbols*

## Abstract

Answers to the title’s question have been influenced by a history in which an early science of consciousness was rejected by behaviourists on the argument that this entails commitment to ontological dualism and “free will” in the sense of indeterminism. This is, however, a confusion of theoretical assertions with metaphysical assertions. Nevertheless, a legacy within computational and information-processing views of mind rejects or de-emphasises a role for consciousness. This paper sketches a mentalistic metatheory in which conscious states are the sole carriers of symbolic representations, and thus have a central role in the explanation of mental activity and action-while specifying determinism and materialism as useful working assumptions. A mentalistic theory of causal learning, experimentally examined with phenomenal reports, is followed by examination of these questions: Are there common roles for phenomenal reports and brain imaging? Is there defensible evidence for unconscious brain states carrying symbolic representations? Are there interesting dissociations within consciousness?

## Introduction

The very title suggests controversy, one that has shaped the aims of psychological science from its founding to the present (Dulany, 2009). I bring a particular perspective, a mentalistic metatheory that I have variously described, (e.g., Dulany, 1997, 2004, 2009).

On that perspective, I will say what I think the roles of conscious states should be in our theories of mental activity and then ask in what ways, given present brain imaging, brain states can have the same and complementary roles. Mental activity is the succession of mental states involved in a range of common phenomena, from perception of the physical and social worlds, through forms of learning and remembering, to reasoning and decision-with infusions of emotion and a sense of self. For any of these, or others (Dulany, 2008), a theory of mental activity would abstract an orderliness among mental states, an orderliness to be empirically evaluated.

## Historically Significant Metatheories

### Early science of consciousness

This controversy over roles for conscious states and brain states started with a critical period in the history of psychology, a period with an enduring influence of a confusion of the theoretical with the metaphysical-which can be seen as a source of conceptual-methodological biases. Psychological science was established as a science of consciousness (Wundt, 1896, 1907) with the *structuralists’ analysis* of conscious states (Titchener, 1898)-a “mental chemistry” that would identify a table of mental elements, and the formulae that would yield any conscious experience, from a sunset to an apple bite. F*unctionalists* turned their focus to the adaptive utility of consciousness, and were strongly guided by the classical work of William James (1890), laying out problems placed within the “stream of consciousness.” *Gestaltists* (Koffka, 1935/1946) focused on the organisational properties of consciousness states, from perception to problem solving. On what today would be called a version of NCC, their principle of “psychophysical isomorphism” held that conscious states and their underlying brain states had a common “functional ordering”-common roles.

### The behavioural revolution–its enduring legacy

Simply put, consciousness was vigorously rejected as a proper subject for science. As John B. Watson put it, “This suggested elimination of states of consciousness as proper objects of investigation in themselves will remove the barrier from psychology which exists between it and the other sciences,” (Watson, 1913/1994, p. 253). The reason? “Behaviourism claims that ’consciousness’ is…merely a word for the ’soul’ of more ancient times. The old psychology is thus dominated by a kind of subtle religious philosophy” (Watson, 1924, p. 3). Furthermore, for a mechanical metaphor, a dominant machine of the time was selected: a Bell Telephone Switchboard. Stimulus “connecting” could produce a muscular “ring,” and so psychological theory could simply be Stimulus-Response Behaviour theory.

With this core commitment, B.F. Skinner (1974) in his most systematic – and enduring – treatment of Radical Behaviourism, called for the rejection of the person as agent, identified with an introspected and nonphysical world. Behaviourists focused their experimental inquiry on rats and pigeons unequipped to report anything behaviourism had rejected. Just rid the science of what Skinner called “the inner man” – and there would be no reason to fear an ontological nonmaterialism or free will in the sense of indeterminism.

That was the enduring confusion. Theoretical assertions are subject to empirical evaluation. Within the science, as we know it, metaphysical assertions are not (Dulany, 2003).

### The cognitive revolution

That legacy? Roediger (2004), writing his column as President of Association of Psychological Science, argued that behaviourism is still very much with us, in journals and societies and behaviour therapy, and in the manipulation – response style of experimental studies throughout psychology. The cognitive revolution was not a renewed focus on consciousness. The relevant legacy has been a rejection of, or de – emphasis upon, a causal role for conscious states and a focus instead on loosely labelled “cognitions” and on claims for complex unconscious processing – claims with experimental support that have been challenged on conceptual and methodological grounds.

Significant factors in the emergence of cognitive metatheories were the following two things: (a) an experimental challenge to Stimulus – Response Behaviour Theory and (b) the emergence of a computer metaphor for mind – the dominant machine of our time. Just think of the mind as a programmed computer and the mind is still a machine after all.

On the *computational view*, (e.g., Jackendoff, 1987), mind and a computer are both instances of a Universal Turing Machine, and so we have the famous analogy: Mind is to brain, as software is to hardware. Most importantly, it is *cognition* that is said to run like a programme in the brain, with consciousness only a sometime and noncausal emergent – epiphenomenalism. So, if consciousness is noncausal, *eliminative materialists*, (e.g., Churchland, 1993), can dismissively consign it to “folk psychology”.

On the *information – processing view*, mind is viewed as a *system* with *subsystems* like those of the computer, and so consciousness is represented as a limited attentional subsystem within a working system – RAM – connected to an LTM and input and output systems, (e.g., Posner, 1994). So, we “store” information in that LTM and then unconsciously “search” and “retrieve,” and unconsciously “compute” outside that attentional subsystem. This metatheory makes a place for consciousness, but it also provides a common commitment to, and emphasis upon, a “cognitive unconscious” (Kihlstrom, 1987) – as we might say, the psychoanalytic unconscious “expurgated.” “Secondary process” becomes “conscious explicit processing” and “primary process” becomes “unconscious implicit processing.”

By far, the most influential for a revival of interest in consciousness has been the Global Workspace view of Baars (1997, p. 43), using the closely analogous theatre metaphor: “…conscious contents are limited to a brightly lit spot of attention onstage…Behind the scenes are executive processes…In the audience are a vast array of intelligent unconscious mechanisms.” Then, with computational modelling of that neurally “global” work – space, (e.g., Baars and Franklin, 2007), the two cognitive metatheories and the newer neurocognitivism blend, the legacy extends.

## Mentalistic Metatheory and Roles for Conscious States

However, viewed more analytically, consciousness is a succession of states, in various modes and contents that have lawful, causal orderliness – to be specified in empirically supportable *theoretical* assertions that in no way entail *metaphysical* assertions of nonmaterial status or free will in the sense of indeterminism. Materialism and determinism are useful *working assumptions*.

### Mental contents

Most significantly, the mentalistic metatheory is set apart in holding that *conscious states are the sole carriers of symbolic representation* – permitting its explanatory power. In consciousness, we represent a present in perception, a past in forms of remembrance, and a future in expectations, intentions, hopes or fears. With higher order representations, we may even represent our own conscious states and mental episodes. Therefore, as we learn what consciousness explains, we enrich the explanation of consciousness.

*Symbols* are functionally specified. They may (a) activate other symbols, e.g., “’café’ activates ’latte,’” and (b) serve as subjects or predicates of propositional contents, as in “A latte is on my desk.” They may (c) participate in the special proposition “’This’ represents ’that,’” e.g., “This ”’latte’ refers to content of ’that cup,’” and (d) in the intention – controlling actions that warrant the preceding proposition: I grasp that cup and sip.

Symbolic contents may be *identity symbols*, attentional identifications of things as such, or *literal symbols* that precede and surround that attentional identification. We attentionally hear what a solo instrument plays from, how it sounds and the surrounding accompaniment.

Symbolic contents may be *propositional*, with *subject* values, such as the relation of what named to what considered or scored correct, and with *predicate* values, such as the likelihood of a certain outcome given the occurrence of what is named in the subject. They may also be *nonpropositional* such as a perception of a trumpet or oboe.

*Modes* are the familiar carriers of conscious contents and vary quantitatively in many experiments. They may be *propositional* modes such as “perceive, or believe, or intend *that* ——,” each carrying a propositional content. Alternatively, they may be *nonpropositional* modes such as a “feeling or only sense *of* ——” for nonpropositional content.

For mental mode and content, we can speak of a sense of *agency*, of possession that may vary in strength and frequency in normalcy, or be diminished in neurosis, and even missing or erroneously attributed in a psychotic syndrome.

### Mental episodes

Mental activity consists of mental episodes – conscious contents interrelated by nonconscious mental operations. In *deliberative mental episodes*, propositional contents are interrelated by deliberative operations; they are decisions or inferences. In *evocative mental episodes*, nonpropositional contents are interrelated by associative – activational operations. We can think of these operations as simply the relations among these conscious states,

$$\mathrm{Cs}\;{\mathrm{State}}_{in+1}\;\leftarrow \;\mathrm{Ncs}\;\mathrm{Op}\;\left(Cs\;State_{jn},..,Cs\;State_{kn-m}\right),$$

sometimes represented by a mathematical function within a model,

$$\mathrm{Cs}\;{\mathrm{States}}_{in+1}\;=\;f\left(Cs\;Stat{\mathrm{es}}_{jn},..,\mathrm{Cs}\;{\mathrm{States}}_{kn-m}\right),$$

– or as the neural processes interrelating the mental states’ coordinate brain states. On this analysis of the commonly but too loosely termed “explicit” or “implicit” (learning, memory, or thinking), these processes are not distinguished by being “conscious” or “unconscious,” but by deliberative and evocative mental episodes (Dulany, 1997).

Forms of *mental episodes are interrelated*. For someone learning to drive, for example, “Red means Stop” can with associative repetition become the evocative episode, “’Red’ activates ’Stop.’” And later, one can readily represent that evocative episode in higher order awareness with the proposition, “Red means Stop.”

The *domain for mental episodes* lies between the final output of *sensory transduction* and the final input to *motor transductions*.

### Higher order awareness

Consciousness may carry symbols representing a past, or even future, conscious state or mental episode (“metacognitively”) by imperfect operations of memory, prediction, or inference. But contrary to Higher Order Thought theories, (e.g., Gennaro, 2004), no mental state on this metatheory must be graced by a higher order state to acquire a “property” of consciousness. That mental state *is* a conscious state.

### The nonsymbolic

*Nonconscious memories* are nonsymbolic neural networks – consistent with that part of connectionism. What we “know,” “believe,” or “intend,” despite a loose vernacular, does not have the same functional specification in inactive memory as in consciousness. Memories in those networks are constructed by experience, not “stored,” and remembering is a process of activation and construction, not “search, identification, and retrieval.”

### Automaticity

In a simple form, one conscious state directly activates another – an evocative episode. Or a conscious intention may be the higher node of a hierarchical structure, activating lower order responses, providing nonpropositional feedbacks for an evocative episode. With automatisation, deliberative thought *drops out* not *down* to an unconscious (Dulany, 1997), consistent with diminished fMRI activity for the controlling network (Chein and Schneider, 2005). Consider a diagnosis that comes automatically to mind from presentation of a familiar set of symptoms.

Some have used aspects of this metatheory explicitly, for example, Carlson (2002), Perruchet and Vinter (2002) and Tzelgov (1997) and others less explicitly in the many studies that examine the roles of conscious states in a range of mental activities – or methodologically challenge various claims for unconscious perception, learning, thinking, etc.

## Mentalistic Theoretical and Experimental Example

A mentalistic theory of propositional learning describes deliberative inferences among a network of conscious beliefs. The learning of causal beliefs was described in a theory refined by a quantitative model (Dulany, 1979), and experimentally applied in identifying the suspect cause of a murder effect in Carlson and Dulany (1988). Subjects were presented two mysteries, each with 12 trials of clues, with different suspects provided 4 different ratios of incriminating and exonerating clues. On each trial, they reported the theory’s belief states, varying from Certain Yes to Certain No (ß = +1 to – 1).

(a) From degree of belief a clue is associated with a suspect, βA – which can vary over suspects, and degree that this clue implies guilt or innocence, the “forward implication,” ßF, one infers subjective evidence, ßE, its implied guilt or innocence for this suspect. That product strongly predicted the subjective evidence.

(b) “Convincingness” of that evidence for that suspect, however, should be the product of subjective evidence for that clue, ßE, and the degree of belief that it would be true or false *only* of the true murderer, the “backward implication,” ßB. Then, from prior belief in this suspect’s guilt – innocence and convincingness of that evidence for this suspect, one may infer a revised belief:

ßH _n+1,i_= ßH _ni_+ /ßE _nij_X ßB _nij_/(1-ßH _in_), if ßE _nij_> 0

ßH _n+1,i_= ßH _ni_, if ßE _nij_= 0

ßH _n+1,i_= ßH _ni,_- /ßE _nij_X ßB _nij_/(1+ßH _in_), if ßE _nij_< 0.

Over all subjects and trials, correlation of predicted and reported causal beliefs was .91, slope of .98, and near zero intercept-with the predictions closely tracking reports over trials for the four different ratios of evidence tending to incriminate or exonerate. There was an explanatory role for conscious states in a causal network.

Examples examining implicit and explicit learning, with no need for mathematical modelling, are Dulany, Carlson and Dewey (1984) and Dulany and Pritchard (2007).

## Comparable Roles for Brain States Underlying Conscious States?

On the assumption that conscious states and brain states are coordinated in some way, underlying brain states could have comparable roles in theories of mental activity. There is an active search for these NCCs, some using invasive methods. For the question I raise, however, where does noninvasive brain imaging with humans stand at its present state of technological development – compared with the utility of phenomenal reports?

### Mechanisms

One example is the search for the neural *mechanisms* underlying symptoms in schizophrenia (e.g., Wibble, Preus and Hahimoto, 2009). Cacioppo and Decety (2009) called for a general programme of identifying the mechanisms underlying more general psychological *processes*, with some focus on use of fMRI. And, the P300 in ERP has long been identified with the general process of “context updating” (Donchin and Coles, 1988).

### Classes of states

Also, well known is fMRI evidence revealing brain areas underlying *classes of conscious states*, e.g., the fusiform face area for facial recognition (Kanwisher, McDermott and Chun, 1997), and a parahippocampal area for place recognition (Epstein and Kanwisher, 1998).

### The challenge of specific states

To some degree, “pattern analysers” facilitate distinguishing more specific states, at least when a controlling stimulus provides the specific state to be identified, for example, vertical or tilted grating, leftward or rightward motion or blue jay or sparrow in Kamitani and Tong (2005). Then, further tests were directed at distinguishing which of competing stimuli were attended – with orientation predicted by activation of sensory areas, V1 – V4 and objects by activation of higher areas.

But could there be a “dictionary” of imaging outputs, something that calls for numerous *identifications?* Discriminating the few with specific training is not equivalent to identifying the many in many contexts over many persons, with control for wandering thoughts. We can also report abstract concepts – “justice” or “energy” – but are they *identifiable* with neuroimaging? I think, too, that propositional forms, those central to deliberative thinking, present a particular problem – with the extraordinarily large combinations of subjects and predicates and degrees of belief or asserted likelihood, as well as evaluations and the sense of ownership of those thoughts.

### General methodological challenges

While recognising significant contributions, Poldrack (2006, 2008) also points to specific limitations, for example, the problem of “reverse inference” from fMRI imaging to localisation of function when the localisation has been identified with various psychological functions. He elaborates especially interesting challenges from psychiatry and advertising (Poldrack, 2009).

In addition, Vul, Harris, Winkiellman and Pashler (2009) identify a large number of studies in which there were unrealistically high correlations between fMRI indices and various personality, emotion and social cognition measures, unreasonably high, given the modest reliability of both measures. They identified many studies in which voxels were selected for computation of their mean values only if the correlations of their own indices – amount of deoxygenated haemoglobin in the blood (the BOLD signal) – with the personality measure met some correlational criterion. This selection of voxel signals artificially reduced unreliability of the fMRI measures, thereby inflating the overall correlations – a statistical violation. Vul and Kanwisher (2010) identify more cases and elaborate the problem, including an especially revealing concluding section entitled “Why the nonindependence error is prevalent in fMRI.“

### A fundamental question

*Viewing these various challenges, I believe a question for neuroimaging technology today is this*: Is there any noninvasive brain imaging method for humans – from ERP and MEG and to the most commonly used fMRI – with the rich variability of output that can *validly* reflect the rich variability of electrochemical activity in the neural networks that must underlie the rich variability of the *specific* states within our mental activity? Magnitude of positive or negative action potentials over a few 100 ms? Distinctive magnetic fields? BOLD signal measures for a pattern of voxels, with the unusual number of complicated steps, as described in Vul and Kanwisher (2010)? The still more recent development of *optical* imaging, including event – related optical signals (Gratton and Fabiani, in press)? *Or must we have still other technological developments?*

## Methodological Strategy for Phenomenal Reporting and Neuroimaging

On a more positive note, brain imaging may reveal brain mechanisms underlying or predisposing various *mental episodes* – those categorised as symptoms, for example, or as deliberative or evocative in general. There could even be a place for imaging of processes underlying specific conscious states with pattern analysis – especially when identified with antecedent stimuli. Brain imaging could also have a particular utility where private reports of those states are unavailable or untrustworthy.

*Confidence in the validity of phenomenal reports and brain images must rely on the same logic within the philosophy of science*. For reasons elaborated in Dulany (2009), reports and brain images would be reported in *data* language, and conscious states and brain states in theory language. On the Duhem – Quine thesis, the hypothesis is an aggregate of Theory, Mappings and Auxiliaries; in Quine’s famous statement “they go to the court of experience as a corporate body.” So, for validation of phenomenal reports or imaging, there are auxiliary conditions to be met and joint support to be obtained from confirming data – competitively so with richness of theory and data. For example, the illustrated theory of causal learning with phenomenal reporting (Carlson and Dulany, 1988) is rich enough to predict data not plausibly explained by traditional associative theories of causal learning.

When the validity of phenomenal neural imaging is challenged, researchers must ask whether there is now a rich enough theory of interrelations among specific brain states, or whether they should rely on theories of interrelations among psychological states.

## Neuroimaging of Unconscious, *Nonsymbolic* States in This Metatheory

Here too, there is growing literature on the neural networks underlying *inactive memories*, as well as *sensory and motor transductions*, to and from conscious states.

## A Challenge From Reported Evidence of Unconscious *Symbolic* States?

Most fundamentally, do these states exist, with consciousness unnecessary for mental activity, and only epiphenomenal when it occurs – thus challenging the adequacy of mentalistic metatheory and scope of phenomenal reporting? Could there then be brain imaging of such states? This cannot be a review of these large and controversial literatures – volition, thinking, learning, perception – but I will focus on some of what I believe are *methodological and conceptual biases* that express the continuing de – emphasis upon consciousness and search for a “cognitive unconscious” – apparent expressions of the grand legacy.

### Unconscious volition?

A major aim in this literature has been the rejection of control of actions by conscious volition – intention, by demonstrating that the “will” is only an epiphenomenon – its control being an illusion.

In a famous series of experiments, Libet (1985) instructed subjects to voluntarily flex their wrist, as many as 40 times – with three critical measures: EMG of the hand movement, a postresponse pointing to a clock to report the time of an “urge to move” (about 200 ms earlier), and a “readiness potential” with EEG that was a negative shift in electric potentiality somewhere in the premotor or motor cortex (about 550 ms earlier). Averaged over trials and subjects, the “readiness potential” precedes the reported “urge” to flex – the intention – will – volition – by 350 ms or so: the “Libet lag.” That “readiness potential” has commonly, though surprisingly, been interpreted as the valid measure of the causally controlling intention – will – volition, permitting the reported urge to be described as merely a noncausal epiphenomenon. That interpretation has been both vigorously endorsed and challenged in various published symposia and books (e.g., Pockett, Banks and Gallagher, 2006).

In particular, a prior “readiness potential” could reflect what its term says – the activation that follows multiple responses and a general decision process, that is, “readiness” to form that more specific intentional urge to respond again. The reported time of intentional urge does precede the response time, and subjects were also able to voluntarily withhold a response. Brass and Haggard (2008) proposed a more complex decision process, reviewing fMRI evidence for “what,” “when” and “whether” states, actually conveyed in this way by the experimental tasks, with variously reported loci, from presupplementary motor area to prefrontal and parietal. These are images that can be interpreted as covering “readiness potential,” decision and conscious intention.

Is Libet’s legacy part of the behaviourist’s legacy? Pockett (2004, p. 624) writes that with “Libet’s main legacy…[we could] perhaps finally solve, the metaphysical debate about free will and determinism.” This, I believe, is the conceptual error that has motivated much of this literature. A theory that actions may be controlled by conscious intentions in no way entails free will in the sense of indeterminism. Intentions have their own causal antecedents and may together be represented in theoretical assertions, empirically investigated on the methodologically useful assumption of determinism – as in a literature extensively developed and reviewed in Ajzen, I. (in press).

A resolution in coordinate models? On the general assumption that conscious states are coordinated with neural states, a theory that *conscious intention* causally controls *observable actions* is coordinated with a theory that their *underlying neural states* causally control *neural response processes*.

### Unconscious thinking – problem solving?

The belief is in the culture, even in anecdotal “examples” provided in undergraduate science classes – Poincaré’s creation of the Fuschian function and Kekulé’s creation of the benzene ring structure. But Ericsson and Simon (1993, p. 162) write, “A critical reading of Poincaré does not provide any evidence for unconscious processing, nor for the belief that Poincaré himself favoured that interpretation.” And the scholarship of Wotiz and Rodofsky (1984, p. 720) debunks the Kekulé story, quoting his own regret for the false claim he made after dinner and alcohol: “Today I have the feeling it would be better if one burned the whole rubbish and did not allow anything to be printed.” As the authors conclude, however, “…we have our doubts that the truth will get in the way of a good story,” (p. 723).

Indeed, there have been vigorous experimental attempts to show an “incubation effect” in problem solving, in solution of anagrams to complex puzzles, defined by this result: If Presentation of Problem is followed by Time Off (with distraction and delay), then performance on the Delayed Test is superior to Presentation followed by Immediate Test. Over the years, the reviewers agree: The “incubation effect” is inconsistently found, and the design is subject to artifacts that can prevent distraction from ruling out conscious problem solving, e.g., “physical refreshment, fruitful forgetting, losing commitment to an ineffective approach, and noticing clues in the environment. Extended unconscious thinking does not occur” (Perkins, 1981, p. 81). And Mandler (1994, p. 220) concludes, “there is no direct evidence that complex unconscious ’work’ (new elaboration and creations of mental contents) contributes to the incubation effects.”

### Unconscious thinking – decision?

With the more recent work, (e.g., Dijkterhuis and Norgren, 2006), there has been a revival of the general claim within a similar paradigm, but with theory focused on decision processes. In one set of experiments, “conscious thought” was identified with the period following a presentation of alternative products – e.g., cars, apartments – and before instructed product evaluation and choice. “Unconscious thought” was then identified with a period of distraction after presentation and before evaluation and choice. The findings emphasised were better choice and postchoice evaluation with “distraction” than with “conscious thought.” In addressing the general metatheoretical matter, Dijksterhuis and Aarts (2010, p. 475) suggest that “One way to approach this issue is to propose that, in principle, the operation of higher cognitive processes does not care much about the conscious state of the individual.” That legacy?

This series of experiments is being met by a burst of experimental critiques – e.g., Lassiter *et al*., 2009; Rey *et al*., 2009; Waroquier *et al*., 2009. In essence, these experiments consist of failures to replicate and the identification of experimental biases. For example, subjects could form their favourable evaluations of familiar attributes on a first presentation – and so could, on ordinary “experimental demand,” revise those evaluations downward. As Lassiter *et al*. (2009, p. 671) refer to their findings, “…such judgments are ultimately a product of conscious rather than unconscious thinking.”

### Unconscious implicit learning?

This is one of the most vigorously investigated and critiqued claims for unconscious symbolic representation. It would consist of unconscious abstraction and representation of guiding and symbolic rules (Reber, 1967) – a claim with many attempts to support and many critical analyses, from Dulany *et al*. (1984/2009) onward, for example, Shanks and St. John (1994), Perruchet and Vinter (2002) and Pothos (2007). I must only list some of the methodological – conceptual biases: (a) “Post experimental” assessment of awareness of sets of rules beyond memory limits. (b) Neglect of correlated and explanatory conscious contents, such as features and exemplars. (c) What is not conscious structural knowledge said to be unconscious knowledg – despite explanation by conscious judgment knowledge. (c) Failure to recognise implicit learning in the sense of establishment of associative – activational (evocative) relations apart from rules – as represented in theory (Dulany, 1997).

### Unconscious perception?

Again, I must only briefly list methodological – conceptual biases: (a) Identification of “subliminal perception,” a disparity between “direct” and “indirect” measures, with “unconscious perception,” its theoretical interpretation, when measurement validity can be challenged. (b) Imperfect stimulus masking recognised from Holender (1968) to Kouider and Dehaene (2007). (c) Use of subjective reports, with susceptibility to criterion bias, as experimentally demonstrated and represented in signal detection theory (Snodgrass, 2002). (d) Relative insensitivity of some objective measures of awareness. (d) Uncontrolled role of unassessed *literal* awareness in producing “subliminal effects” (Dulany, 2001). With these and other limitations of this literature, I can agree with Merikle and Reingold (1998, p. 309), early and influential proponents of unconscious perception, who wrote, “We doubt that it will be possible ever to prove the existence of unconscious perception.”

## Some More Positive Implications: Dissociations *Within* Consciousness

Consistent with prevailing metatheoretical views, two well – known syndromes have been interpreted as cases of chronic unconscious perception, but the phenomena may be more confidently and productively interpreted within mentalistic theory as dissociations *within* awareness.

### Prosopagnosia

With damage to a region of the fusiform gyrus, the patient may be unable to recognise faces consciously, yet nevertheless respond systematically in various ways to the person’s face. For example, Tranel and Damasio (1985) found greater GSR to familiar than unfamiliar faces. Furthermore, DeHaan *et al*., (1992) even found that classifying a name as politician or nonpolitician was slowed when the face was accompanied by a face from the opposite category. On the standard explanation, (e.g., Young, 1994), this is *unconscious facial recognition*, a dissociation between consciousness and a facial recognition system.

*The key to a mentalistic explanation is recognition of a role for literal awareness dissociated from identity awareness*. Identity awareness is blocked when activation fails to reach temporal areas, but literal awareness of face can activate other neural networks, producing a GSR to a familiar face, as in Tranel and Damasio (1985), and activating various occupational associations incompatible with the erroneous labels, as in DeHaan *et al*. (1992). Is the mentalistic explanation more tenable and promising? Literal facial forms are definitely represented in the patient’s awareness, activating other networks, as shown in their normal ability to match facial photos, familiar or not (DeHaan *et al*., 1987). With Farah (1994), this agrees that the recognition system is damaged, but the remaining parts produce these effects.

### Blindsight

What has spawned this Oxonian oxymoron? With damage to striate cortex, V1, some subjects, most famously the Oxonian subjects DB and GY, showed significant discrimination of stimuli presented to the scotoma, despite lack of, to quote Weiskrantz (1997, p. 65), “any awareness whatsoever of a visual event” – and I would emphasise the last two words. In the more advanced procedures, the subject significantly labels the direction of a moving figure, to the right or left, but reports Yes or No with what is termed a “commentary key,” nonsignificantly as to whether this experience is something they would characterise as visual, as seeing. On the standard interpretation, this is compelling evidence of a dissociation of consciousness from perception – unconscious perception.

*The key to a mentalistic explanation is in recognising that lower order awareness may be dissociated from a higher order awareness*. We need to first recognise two biases in the standard interpretation. (a) What is said to tap the unconscious perception is simply what has long been recognised, from signal detection theory (SDT) and Merikle and Reingold (1998), as the most sensitive measure of awareness of a stimulus – the direct objective. A significant d’ for that index is obtained when clearly conscious assertions as to the direction of movement are significantly associated with actual direction of movement. (b) Furthermore, with use of the “commentary key,” the subject *categorises* that just prior experience as visual, as seeing, or not – a content of a *higher order awareness*. In a revealingly entitled paper, “Varieties of residual experience,” Weiskrantz (1980) wrote of EY that “when he was asked to report when he *saw* the light coming into his field – he was densely blind by this criterion, but when he was asked to report merely when he was *aware* of something coming into his field, the field was practically filled” (p. 378). Working with another subject, Overgaard *et al*. (2009) recently report an interesting continuous relationship between accuracy of her discrimination and a Perceptual Accuracy Scale, a scale of that higher order awareness.

Awareness too degraded to be categorised as “seeing” or varying in degree to which it could be so categorised, could be explained by residual activation of V1 or transmission from the retina that bypasses the dorsal lateral geniculate route to V1, going instead through the superior colliculus to other parts of the visual system, V2, V3, V4 – alternative routes that Weiskrantz (1997, p. 128) acknowledges.

More generally, we can recognise the variety of ways we characterise our conscious experience in higher order awareness, and this yields a variety of questions.

## Conclusions [see also Figure 1]

**Figure 1:**
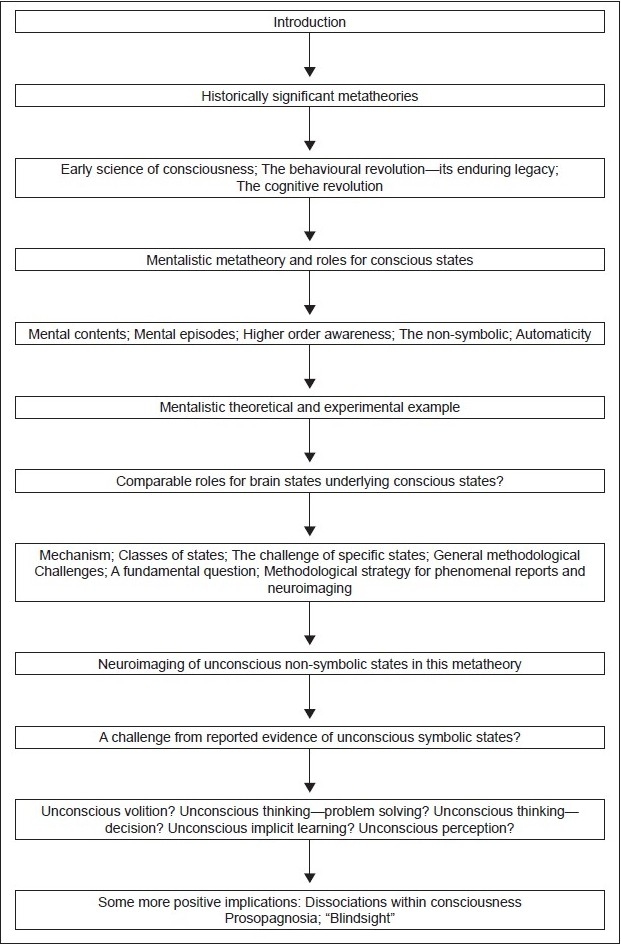
Flow chart of paper

Together, these conclusions constitute what might be called a “take – home message”:

1. The behaviourist rejection of a science of psychology was animated by two intellectual confusions: A theoretical role for consciousness entails ontological immaterialism and “free will” in the sense of indeterminism.
2. With the cognitive revolution, the computational metatheory accorded consciousness the role of an epiphenomenon, and the information processing metatheory emphasised intelligent processing that was symbolic but unconscious.
3. A mentalistic metatheory, however, holds that conscious states are sole carriers of symbolic representation, and mental episodes interrelate propositional or sub – propositional contents with nonconscious operations, deliberative or associative – activational – the essence of mental activity.
4. For validation, phenomenal reports and brain imaging call on the same logic within the philosophy of science – joint empirical support of mapping and theoretical hypotheses.
5. Brain imaging can be very useful, but phenomenal reporting and brain imaging would serve somewhat different roles in its present state of technological development.
6. In studies of volition, thinking, learning and perception, I do not find methodologically acceptable evidence that would challenge a mentalistic metatheory and call for brain imaging of unconscious symbolic states.

## Questions That This Paper Raises

The most fundamental, of course, is in the title – and addressed throughout the paper. But addressing controversial questions inevitably raises further questions:

1. How productive will a mentalistic metatheory be in implying theories that are experimentally supported – as was the illustrated theory of causal learning?
2. How soon can we expect technological advances that will permit brain imaging to identify specific states that are stimulus independent, abstract, and/or propositional?
3. Can we expect conceptual and methodological rigour to overcome, more generally, what I have characterised as the legacy of behaviourism?
4. And on that more positive question: How productive can we expect a turn of interest to dissociations within consciousness to be – between literal and identity awareness, between lower and higher order awareness?

## About the Author

*Donelson Dulany received his PhD in psychology from the University of Michigan and is now Professor of Psychology Emeritus at the University of Illinois where his teaching has included graduate courses in consciousness and the nonconscious, as well as in philosophy of science for methodology in psychology. From 1988 into 2009, he was Editor of the American Journal of Psychology. His work has consisted of experimental and theoretical analyses of the roles of conscious states in sources of intentional action, causal reasoning and explicit and implicit learning. This has led to a metatheoretical view, termed ‘mentalism,’ in which conscious states are the sole carriers of symbolic representation within mental episodes that can be deliberative or associative – activational. He is a member of Psychonomic Society and Association for Scientific Study of Consciousness and is a Fellow of American Psychological Association and Association for Psychological Science. His listings include Marquis Who’s Who in America and Marquis Who’s Who in the World.*

## References

[1] Ajzen I, van Lange P.A, Kruglanski A.W, Higgins E.T (in press), A theory of planned behavior: A reasoned action approach. *Handbook of Theories of social psychology*.

[2] Baars B.J (1997). *In the theater of consciousness*.

[3] Baars B.J, Franklin S (2007). An architectural model of conscious and unconscious brain functions: Global Workspace Theory and IDA, *Neural Networks*.

[4] Brass M, Haggard P (2008). ‘The what, when, whether model of intentional action’. *The Neuroscientist*.

[5] Caciopo J.T, Decety J (2009). What are the brain processes on which psychological processes are based?. *Current Directions in Psychological Science*.

[6] Carlson R.A, Ross B Conscious intentions in the control of skilled mental activity. *The psychology of learning and motivation*.

[7] Carlson R, Dulany D (1988). Diagnostic reasoning with circumstantial evidence, *Cognitive Psychology*.

[8] Chein J, Schneider W (2005). Neuroimaging studies of practice-related change in fMRI and meta-analytic evidence of a domain-general network for learning, *Cognitive Brain Research*.

[9] Churchland P, Goldman A.I (1993). ‘Eliminative materialism and the propositional attitudes’, In *Readings in philosophy and cognitive science*.

[10] Dehaan E.H, Young A, Newcombe F (1987). Face recognition without awareness, *Cognitive Neuropsychology*.

[11] Dehaan E.H, Bauer R.M, Greve K.W (1992). Behavioural and physiological evidence for covert face recognition in a prosopagnosic patient, *Cortex*.

[12] Dijksterhuis A, Nordgren LF (2006). A theory of unconscious thought, *Perspectives on Psychological Science*.

[13] Dijksterhuis A, Aarts H (2010). Goals, attention, and (un)consciousness, *Annual Review of Psychology*.

[14] Donchin E, Coles M (1988). Is the P300 component a manifestation of context updating? *Behavioral and Brain Sciences*.

[15] Dulany D (1979). A theory of propositional learning. Unpublished manuscript, University of Illinois.

[16] Dulany D, Cohen J, Schooler J (1997). Consciousness in the explicit (deliberative) and implicit (evocative). *Scientific approaches to consciousness*.

[17] Dulany D.E (2001). Inattentional awareness. *Psyche: An Interdisciplinary Journal of Research on Consciousness*.

[18] Dulany D.E (2003). Strategies for putting consciousness in its place, *Journal of Consciousness Studies*.

[19] Dulany D.E, Gennaro R.J (2004). Higher order representation in a mentalistic metatheory. *Higher order thought theories of consciousness*.

[20] Dulany D.E (2008). How well are we moving toward a most productive science of consciousness?. *Journal of Consciousness Studies*.

[21] Dulany D.E, Bayne T, Cleeremans A, P Wilken (2009). Psychology and the study of consciousness. *The Oxford Companion to Consciousnes*.

[22] Dulany D.E (2009). (1984/2009), A case of syntactic learning and judgment: How conscious and how abstract? Journal of Experimental Psychology: General. *Psychology of learning*.

[23] Dulany D.E, Pritchard E (2007). Awareness and novelty in explicit (deliberative) and implicit (evocative) learning and memory of a finite state grammar. Association for the Scientific Study of Consciousness, Online.

[24] Epstein R, Kanwisher N (1998). A cortical representation of local visual environment, *Nature*.

[25] Ericsson K.A, Simon H.A (1993). *Protocol analysis: Verbal reports as data*.

[26] Farah M, Umilta M, Moscovitch M (1994). Visual awareness and visual perception after brain damage: A tutorial overview. *Attention and performance. Conscious and nonconscious information processing*.

[27] Gennaro R.J (2004). *Higher order thought theories of consciousness*.

[28] Gratton G, Fabiani M Trends in Cognitive Science. Shedding light on brain function. *The event related optical signal*.

[29] Holender D (1986). Semantic activation without conscious identification in dichotic listening, parafoveal vision and visual masking, Behavioral and Brain Sciences.

[30] Jackendoff R (1987). Consciousness and the computational mind.

[31] James W (1890). *Principles of psychology*.

[32] Katamitani Y, Tong F (2005). Decoding the visual and subjective contents of the human brain. *Nature Neuroscience*.

[33] Kanwisher N, McDermott J, Chun M.M (1997). The fusiform face area: A module in human extrastriate cortex specialized for face perception. *Journal of Neuroscience*.

[34] Kihlstrom J (1987). The cognitive unconscious. *Science*.

[35] Koffka W (1963). *Principles of Gestalt psychology*.

[36] Kouider S, Dehaene S (2007). Levels of processing during non-conscious perception. a critical review of visual masking, *Philosophical Transactions of the Royal Society*.

[37] Lassiter G.D, Lundberg M.J, Gonzalez-Vallejo, Bellezza F.S, Phillips N.D (2009). The deliberation-without-attention effect. *Psychological Science*.

[38] Libet B (1985). Unconscious cerebral initiative and the role of conscious will in voluntary action. *Behavioral and Brain Sciences*.

[39] Mandler G, Umiltà C (1994). Hypermnesia, incubation, and mind popping: On remembering without really trying. *Attention and Performance (XV, 34)*.

[40] Merikle P.M, Reingold E.M (1998). On demonstrating unconscious perception. Comment on Draine and Greenwald (1998), *Journal of Experimental Psychology*.

[41] Overgaard M, Fehl K, Mouridsen K, Bergholt B, Cleeremans A (2008). *Seeing without seeing? Degraded conscious vision in a blindsight patient, PLoS One*.

[42] Perkins D.N (1981). *The mind’s best work*.

[43] Perruchet P, Vinter A (2002). *The self-organizing consciousness, Behavioral and Brain Science*.

[44] Pockett S (2004). *Hypnosis is the death of “subjective backward referral,” Consciousness and Cognition*.

[45] Pockett S, Banks W.P, Gallagher S (2006). *Does Consciousness Cause Behavior?*.

[46] Poldrack R (2006). Can cognitive processes be inferred from neuroimaging data?. *Trends in Cognitive Science*.

[47] Poldrack R (2008). The role of fMRI in Cognitive neuroscience: where do we stand?. * Current Opinion in Neurobiology: Science Direct*.

[48] Poldrack R (2009). Neuroimaging: Separating the promise from the pipe dreams, Cerebrum. *The Dana Foundation*.

[49] Posner M.I (1994). Attention. *The mechanisms of consciousness, Proceedings of the National Academy of Sciences*.

[50] Pothos E (2007). *Theories of artificial grammar learning, Psychological Bulletin*.

[51] Reber A.S (1967). *Implicit learning and artificial grammars, J Verbal Learn Verbal Behav*.

[52] Rey A, Goldstein R.M, Perruchet P (2008). *Does unconscious thought improve complex decision making? Psychological Research*.

[53] Roediger H.L (2004). *What happened to behaviorism? The Observer*.

[54] Shanks D.R, St.John M.F (1994). *Characteristics of dissociable human learning Systems, Behavior and Brain Sciences*.

[55] Skinner B.F (1974). *About behaviorism*.

[56] Snodgrass M (2002). *Disambiguating conscious and unconscious influences: Do exclusion paradigms demonstrate unconscious perception? Am J Psychol*.

[57] Titchener E.B (1898). *The postulates of a structural psychology, Philosophical Review*.

[58] Tzelgov J, Wyer R.S, Srull T.S (1985). Automatic but conscious: That is how we act most of the time. *Advances in social cognition*.

[59] Tranel D, Damasio A.R (1985). Knowledge without awareness. *An autonomic index of facial recognition by prosopagnosiacs, Science*.

[60] Vul E, Harris C, Winkielman P, Pashler H (2009). *Puzzlingly high correlations in fMRI studies of emotion, personality, and social cognition, Perspectives in Psychological Science*.

[61] Vul E, Kanwisher N (2010). The non-independence error in fMRI data analysis. *Foundations and philosophy for human neuroimaging*.

[62] Young A.W, Umiltà C, Moscovitch M (1994). Conscious and nonconscious recognition of familiar faces. *Attention and performance*.

[63] Waroquier L, Marchiori D, Klein O, Cleeremans A (2009). *Methodological pitfalls of the unconscious thought paradigm, Judgment and Decision Making*.

[64] Watson J.B (1913/1994),Psychology as the behaviorist views it. *Psychological Review*.

[65] Watson J.B (1924). *Behaviorism*.

[66] Weiskrantz L (1980). Varieties of residual experience. (Eighth Sir Frederic Bartlett Lecture). *Quarterly Journal of Experimental Psychology*.

[67] Weiskrantz L (1980). *Consciousness lost and found*.

[68] Wible C.G, Preus A.P, Hahimoto R (2009). A cognitive neuroscience view of schizophrenic symptoms: Abnormal activation of a system for social perception and communication. *Brain Imaging and Behavior*.

[69] Wotiz J, Rudofsky S (1984). Kekulé’s dreams: Fact or fiction?. *Chemistry in Britain*.

[70] Wundt W, Judd C.H (1896, 1907), *Grundriss der Psychologie*. *Outline of Psychology*.

